# First microscopic, pathological, epidemiological, and molecular investigation of *Leucocytozoon* (Apicomplexa: *Haemosporida*) parasites in Egyptian pigeons

**DOI:** 10.3389/fvets.2024.1434627

**Published:** 2024-08-07

**Authors:** Ismail Saad Elshahawy, Eman Sayed Mohammed, Amany Sayed Mawas, Dina M. W. Shibat El Hamd, Esraa Ali, Abeer M. Alghamdi, Hind Alzaylaee, Ehab Kotb Elmahallawy

**Affiliations:** ^1^Department of Parasitology, Faculty of Veterinary Medicine, South Valley University, Qena, Egypt; ^2^Department of Pathology and Clinical Pathology, Faculty of Veterinary Medicine, South Valley University, Qena, Egypt; ^3^Department of Poultry Diseases, Animal Health Research Institute (AHRI), Agricultural Research Center (ARC), Qena, Egypt; ^4^Department of Parasitology, Animal Health Research Institute, (AHRI), Agricultural Research Center (ARC), Qena, Egypt; ^5^Department of Biology, Faculty of Science, Al-Baha University, Al-Baha, Saudi Arabia; ^6^Department of Biology, College of Science, Princess Nourah bint Abdulrahman University, Riyadh, Saudi Arabia; ^7^Departamento de Sanidad Animal, Grupo de Investigación en Sanidad Animal y Zoonosis (GISAZ), Universidad de Córdoba, Córdoba, Spain; ^8^Department of Zoonoses, Faculty of Veterinary Medicine, Sohag University, Sohag, Egypt

**Keywords:** *Leucocytozoon*, pigeon, Egypt, epidemiology, molecular, phylogenetic, histopathology

## Abstract

**Introduction:**

*Leucocytozoon* is an intracellular blood parasite that affects various bird species globally and is transmitted by blackfly vectors. This parasite is responsible for leucocytozoonosis, a disease that results in significant economic losses due to reduced meat and egg production. There is limited knowledge about the epidemiological pattern of leucocytozoonosis and its causative species in Egypt, particularly in pigeons.

**Methods:**

The current study involved the collection of 203 blood samples from domestic pigeons from various household breeders and local markets across Qena Province, Upper Egypt. Samples were initially examined for potential *Leucocytozoon* infection using blood smears, followed by an evaluation of associated risk factors. Molecular identification of the parasite in selected samples (n = 11), which had initially tested positive via blood smears, was further refined through nested PCR and sequence analysis of the mitochondrial cytochrome b gene to ascertain the *Leucocytozoon* species present. Additionally, histopathological examination of the liver, spleen, and pancreas was conducted on animals that tested positive by blood smears.

**Results:**

Interestingly, 26 out of 203 samples (12.08%) had confirmed *Leucocytozoon* infections based on microscopic analysis. Additionally, all 11 samples that initially tested positive via blood smears were confirmed positive through nested PCR analysis, and their sequencing revealed the presence of *Leucocytozoon sabrazesi*, marking the first report of this parasite in Egypt. The study into potential risk factors unveiled the prevalence of *Leucocytozoon* spp. seems host gender-dependent, with males exhibiting a significantly higher infection rate (33.33%). Additionally, adult birds demonstrated a significantly higher infection prevalence than squabs, suggesting an age-dependent trend in prevalence. Seasonality played a significant role, with the highest occurrence observed during summer (37.25%). Histopathological examination revealed the presence of numerous megaloschizonts accompanied by lymphocytic infiltration and multiple focal areas of ischemic necrosis.

**Conclusion:**

To our knowledge, this is the first study to shed light on the epidemiological characteristics and molecular characterization of leucocytozoonosis in pigeons in Egypt. Further research endeavors are warranted to curb the resurgence of *Leucocytozoon* parasites in other avian species across Egypt, thereby refining the epidemiological understanding of the disease for more effective control and prevention measures.

## 1 Introduction

Pigeons are abundant and ubiquitous avian species, often found in urban environments. Since ancient times, pigeons have been regarded as symbols of various concepts, including deities, peace, messengers, pets, food, and spiritual sacrifice. In Egypt, pigeons are primarily raised to meet the protein needs of families during special occasions, serve as a source of income, for gaming, and ornamental purposes. However, pigeons can host numerous pathogens and serve as reservoirs for parasitic infections (1). Parasitism is a significant concern affecting bird production, leading to issues such as growth retardation, decreased vitality, blood loss, toxicosis, and poor health conditions. Ultimately, this reduces the quality and quantity of meat and egg production. Among various parasitic diseases affecting avian species, haemoprotozoan infections are predominant (2).

Avian haematozoa comprise a class of vector-borne parasites belonging to the apicomplexan group, which includes genera such as *Plasmodium*, *Haemoproteus*, and *Leucocytozoon*. These parasites are transmitted by blood-sucking dipteran vectors, including species of ceratopogonids (genus *Culicoides*), blood-sucking culicine mosquitoes (*Culicidae*), blackflies (*Simuliidae*), and hippoboscid flies, with birds acting as intermediate hosts (2). Among others, leucocytozoonosis is considered the most significant blood protozoan disease affecting birds, caused by approximately 60 species of parasitic protozoa of the genus *Leucocytozoon*. It affects wild and domestic avian species and is transmitted by biting blackflies such as *Simulium venustum*, *S. croxtoni*, *S. euradminiculum*, and *S. rugglesi* (3). The life cycle of *Leucocytozoon* species is complex, involving development both in tissues (exo-erythrocytic merogony) and blood cells. Before infecting blood cells and forming gametocytes, *Leucocytozoon* species undergo exo-erythrocytic merogony, which produces meronts in tissue cells. These meronts are the infective stage for vectors. Subsequently, sexual processes and sporogony occur in dipteran insects, producing infective sporozoites, which initiate new infections in vertebrate hosts (2, 4, 5). The pathogenic impact of *Leucocytozoon* infection on the host can potentially jeopardize productivity, reducing egg production and increasing mortality rates. Clinical signs of infection may include anemia, anorexia, green feces, and ataxia, although infections can be asymptomatic. Upon necropsy, common findings include fatty liver, splenomegaly, regressive reproductive organs, and other characteristic lesions. This can result in a significant loss of production value in industrial settings and group deaths, as reported in various avian species (6, 7).

Direct microscopic examination of Giemsa-stained blood films was considered the most conservative diagnostic approach for detecting *Leucocytozoon* sp. infection. Additionally, identifying the parasite’s genome using polymerase chain reaction (PCR) with primers derived from mitochondrial genes offers a more sensitive and accurate method widely employed in laboratory settings for precise analysis of infections. This molecular approach can provide high accuracy even in cases where blood smears are negative due to low parasitemia or early stages of infection in avian hosts (8–10). Understanding the epidemiological patterns of parasitic infections is essential for devising and implementing effective prevention and control strategies. Investigating the previous literature, very scant information is available on Egypt’s *Leucocytozoon* sp. infection. Only one previous study in northern Egypt (lower Egypt) revealed the natural co-infection of poultry farms with *Leucocytozoon caulleryi* and chicken anemia virus (11). However, to the best of our knowledge, no specific investigations have been conducted to explore the incidence of blood parasitic infections, particularly *Leucocytozoon* species, in the country’s southern region (Upper Egypt). Therefore, the current study was conducted to identify and determine the prevalence of *Leucocytozoon* species in pigeons from Upper Egypt and assess the associated risk factors through microscopic examination of stained blood smears. Additionally, this investigation examined the taxonomy of the identified leucocytozoids at the species level by analyzing the phenotypic characteristics of the cytochrome b gene (*cytb*) and reporting the major histopathological findings of the examined animals.

## 2 Materials and methods

### 2.1 Ethical statements

The present study received approval from the Ethics Committee of the Faculty of Veterinary Medicine at South Valley University, Egypt, per ethical regulations and animal research guidelines (permit code No. 84). Written and oral consent was obtained from each owner of the surveyed pigeons.

### 2.2 Study area

The study was conducted in Qena Province, situated in southern Egypt at coordinates 26°10′12″N 32°43′38″E. Renowned for its pottery, imposing mountains, and lush green landscapes, the province experiences a hot desert climate characterized by scorching summers and minimal yearly precipitation.

### 2.3 Birds and sample processing

Blood samples (*n* = 203) were randomly collected from apparently healthy pigeons in Qena Province between November 2020 and October 2021, sourced from different household breeders and local markets. Information on age, sex, and sampling season was documented to evaluate potential associations with the presence and abundance of blood parasites. Each bird’s sample (3 mL) was gathered in an anticoagulated test tube from the brachial wing vein using a sterile syringe and needle. These samples were transported to the Parasitology Laboratory at the Faculty of Veterinary Medicine, South Valley University, for parasitological analysis.

### 2.4 Laboratory analysis

Following collection, thin blood films were immediately prepared from each sample to identify blood protozoa. The smears were air-dried, fixed in absolute methanol three times for 10 s each, and stained with Giemsa’s stain (30%) for 10 min. Subsequently, the slides were gently washed under running tap water, air-dried, and then subjected to microscopic examination, following established laboratory protocols (12). The stained slides were examined using an Olympus CX31 microscope at higher magnification (100X) to detect infections. The identification and intensity of recovered haemoprotozoa were recorded following established keys and descriptions outlined by Soulsby (13) and Levine (14).

### 2.5 Histopathological analysis

The examined pigeons were anesthetized using an equal mixture of ketamine and xylazine (0.0044 cc/kg), administered via injection into the pectoral muscle (15), then left to ensure complete euthanasia. Tissue samples, mainly liver, spleen, and pancreas, were then excised and prepared for histopathological examination (16). Approximately 1 cm sections of each tissue were collected and fixed in 10% neutral buffered formalin (pH = 7.4). They underwent processing through ascending grades of alcohols, were embedded in paraffin wax, sectioned at a thickness of 5 μm, and then stained with histochemical stains (Harries hematoxylin and eosin, Sigma-Aldrich) (17). The preparations were examined using a microscope (Olympus BX51, Tokyo, Japan) equipped with a camera (Olympus E-182330, Olympus Optical Co., Ltd., Japan), with five slides inspected for each block.

### 2.6 Molecular identification

#### 2.6.1 DNA extraction

DNA was extracted successfully from 11 positive blood samples via microscopic examination. This extraction was performed using a QIAamp DNA mini kit (1,043,368, Qiagen, United States) following the manufacturer’s instructions, and the extracted DNA was stored at −20°C until PCR analysis.

#### 2.6.2 PCR amplification

Two pairs of specific primers from Macrogen (Korea) were utilized to amplify the *cytb* gene via nested PCR. The first step of amplification employed the primers LsF1 (5′-CATATATAAGAGAATTATGGAG-3′) and LsR1 (5′-ATAAAATGYTAAGAAATACCATTC-3′). In the second step, the primers LsF2 (5′-TAATCACATGGGTTTGTGGA-3′) and LsR2 (5′-GCTTTGGGCTAAGAATAATACC-3′) were utilized. The expected size of amplification products was 248 bp. The reaction was conducted in a 25 μL volume containing 12.5 μL of DreamTaq Green PCR Master Mix (2X) (K1081, ThermoFisher, United States), 1 μL of each primer (20 pmol), 5.5 μL of water, and 5 μL of DNA. PCR was performed using an Applied Biosystems 2,720 thermal cycler with the following program: 40 cycles of denaturation at 94°C for 1 min (1st step) and 20 s (2nd step), annealing at 50°C for 1 min (1st step) and 53°C for 20 s (2nd step), extension at 68°C for 1 min (1st step) and 30 s (2nd step), followed by a final extension at 68°C for 5 min (1st and 2nd steps).

#### 2.6.3 Visualization of the PCR outcomes

The PCR products were subjected to electrophoresis on a 1.5% agarose gel (Agarose, Universal, PeqGold, Peqlab. Germany) in 1x TBE buffer. About 20 μL of the PCR products was loaded onto the gel. A 50 bp DNA ladder gene marker (PeqGold 2 kb DNA-Ladder, Peqlab., VWR) was utilized to determine the size of the amplicons. The gel was stained with ethidium bromide (0.5 μg/mL) and visualized under UV light. The results were documented using a gel documentation system (Geldoc-it, UVP, England).

#### 2.6.4 Sequencing and phylogenetic analysis

The PCR products were purified using a QIAquick PCR product extraction kit (Qiagen, Valencia) and Centrisep spin columns. The gel documentation system (Geldoc-it, UVP, England) was utilized to capture the sequence reaction, and analysis was conducted using Totallab analysis software.^1^ The identity of the obtained DNA sequences from the ABI PRISM® 3,100 Genetic Analyzer (Micron-Corp. Korea) was confirmed through BLAST analysis (18). The phylogenetic tree was constructed using the MegAlign module of Lasergene DNA Star version 1.83 software, based on the *cytb* gene sequences (19). The analysis was conducted in MEGA11 using the accession number (ON399180) with maximum likelihood method (20). The sequences, along with their corresponding host and locality or country, were downloaded from GenBank and used in the tree construction, as depicted in Table 1.

**Table 1:** The accession numbers used for construction of the phylogenetic tree, along with their species, hosts, and locations.

Accession number	Species	Host origin	Location/country
MW316431	*Leucocytozoon sabrazesi*	Chicken	Thailand
MW316432	*Leucocytozoon sabrazesi*	Chicken	Thailand
MW316434	*Leucocytozoon sabrazesi*	Chicken	Thailand
KT290930	*Leucocytozoon sabrazesi*	*Gallus gallus spadiceus*	Malaysia
MZ634390	*Leucocytozoon sabrazesi*	*Gallus gallus*	Thailand
KT290929	*Leucocytozoon sabrazesi*	*Gallus gallus domesticus*	Malaysia
AB299369	*Leucocytozoon sabrazesi*	Chicken	Malaysia
LC550031	*Leucocytozoon sabrazesi*	Chicken	Myanma
MW600919	*Leucocytozoon caulleryi*	*Gallus gallus*	Thailand
MN540144	*Plasmodium kentropyxi*	Lizards	Brazil
MW296834	*Haemoproteus* sp.	Avian	Korea
JQ988310	*Parahaemoproteus* sp.	*Coeligena torquata*	Peru
MK721052	*Leucocytozoon* sp.	*Emberiza godlewskii*	China
MK061720	*Haemoproteus* sp.	*Pachycephala hyperythra*	Papua New Guinea
GU59370	*Eimeria acervulina*	*Gallus gallus*	United States

### 2.7 Statistical analysis

Statistical analysis was conducted to assess the variation in *Leucocytozoon* spp. incidence among pigeons, considering the epidemiological data. The chi-square (χ^2^) test was employed using IBM SPSS Statistics for Windows, Version 21.0 (IBM Corp., Armonk, NY, United States). A significance level of *p* ≤ 0.05 was considered indicative of statistical significance (21).

## 3 Results

### 3.1 Occurrence of *Leucocytozoon* species and potential risk factors

Out of 203 inspected pigeons, 26 were infected with *Leucocytozoon* species, yielding an overall prevalence of 12.80%. Regarding infection frequency (Table 2), *Leucocytozoon* spp. showed significantly higher rates in the age group of >2 months (22.2%, *χ^2^* = 19.063, *p* < 0.05), while the other age category remained unaffected, suggesting that the risk of infection rises with age. Likewise, the current investigation revealed that the occurrence percentage of *Leucocytozoon* spp. was 10.43% in females, whereas it was 33.33% in males, indicating a statistically significant gender disparity in infection rates (*χ^2^* = 8.836, *p* = 0.002) as depicted in Table 2. Additionally, the same table presents the prevalence of parasite infections relative to both age and sex groups in examined pigeons. It is intriguing that the proportion the occurrence rate of *Leucocytozoon* infection was notably higher in males (46.66%) compared to females (18.62%) within the same age category (>2 months), which demonstrated a statistically significant difference (*χ^2^* = 13.079, *p* = 0.004). Conversely, no infections were observed in either male or female pigeons in the younger class (<2 months).

**Table 2:** Prevalence of *Leucocytozoon* spp. in relation to the age, sex, and season of the pigeons inspected in the present study.

Variables	No. of examined cases	No. of positive cases (%)	Pearson Chi-Square *χ*^2^ (*p* value)
Age			19.063 (< 0.0001)*
< 2 months	86	0 (0)	
> 2 months	117	26 (22.22)	
Sex			8.836 (0.002)*
Male	21	7 (33.33)	
Female	182	19 (10.43)	
Season			36.593 (<0.05)*
Winter	47	0 (0)	
Spring	52	6 (1.92)	
Summer	51	19 (37.25)	
Autumn	53	1 (1.88)	

*Superscript indicates the significant difference at *p* < 0.05.

Investigation of seasonal dynamic of infection with *Leucocytozoon* species showed that the summer season exhibiting the highest prevalence of *Leucocytozoon* infections (37.25%), followed by spring (1.92%) and autumn (1.88%). No infection was observed during the winter (0%), indicating a notable difference in infection seasonality, as shown in Table 2. Additionally, the variations in infection rates between seasons were statistically significant (*χ*^2^ = 36.593, *p* < 0.05).

### 3.2 Morphological description

**Figure 1:**
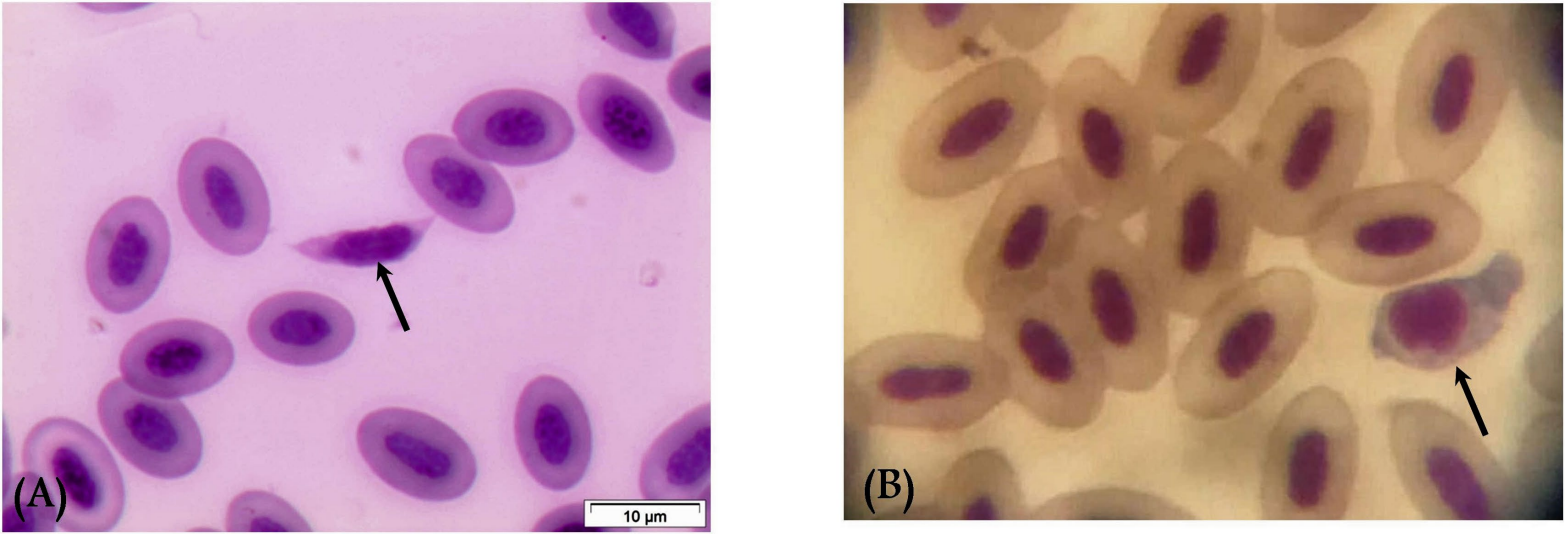
Mature macrogametocyte **(A)** and microgametocyte **(B)** of *Leucocytozoon sabrazesi* (arrows).

**Figure 2:**
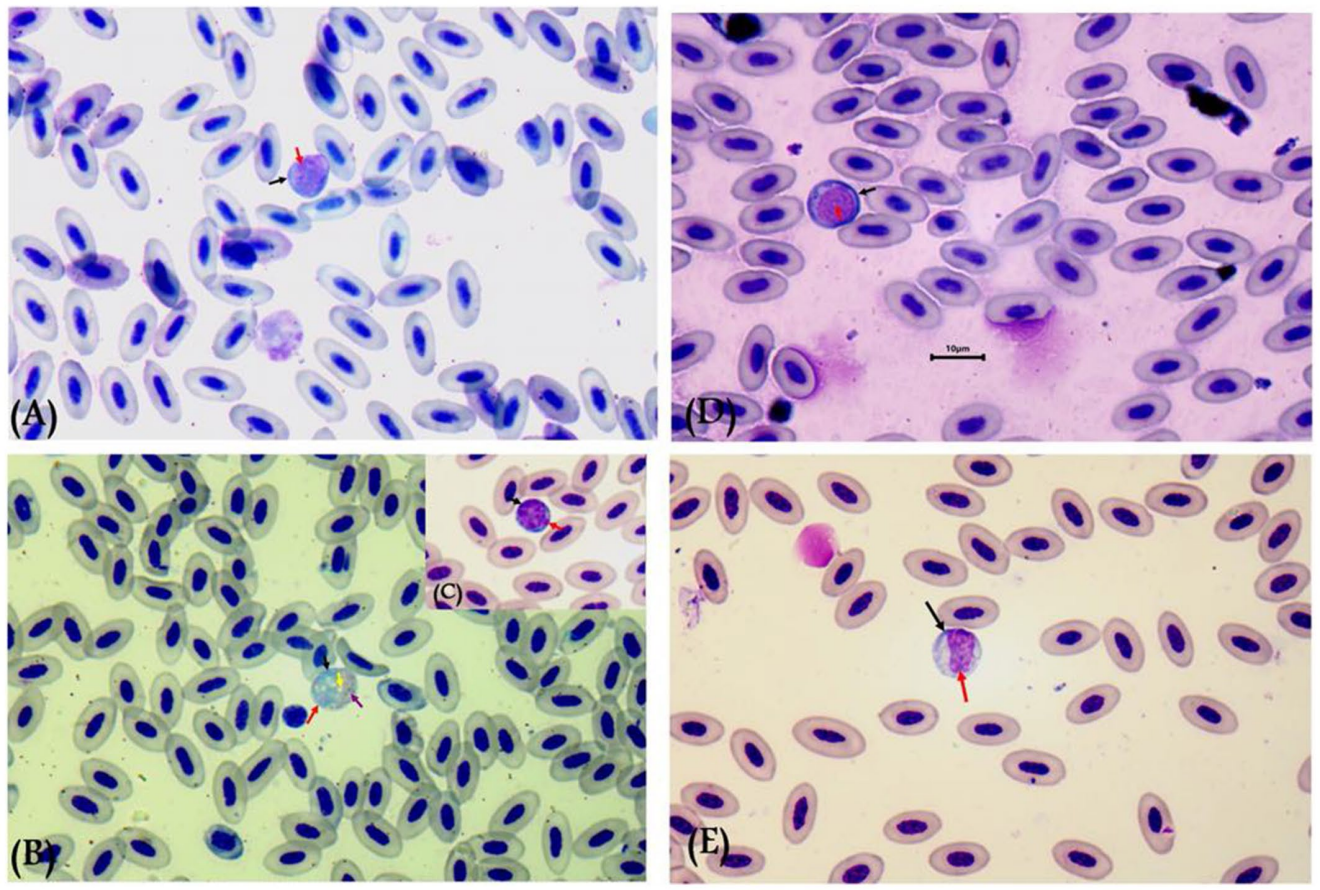
*Leucocytozoon sabrazesi* macrogametocytes **(A–C)** and microgametocytes **(D,E)** in pigeon’s cells. **(A,E)** gametocytes in elongated form and **(C,D)** gametocytes in round form in pigeon’s leukocyte cells **(C,D)**. The black arrow indicates host cell nuclei, the red arrow indicates nuclei of parasites, the yellow arrow indicates vacuoles, and the violet arrow indicates volutin granules. Scale bar = 10 μm.

Gametocytes of *L. sabrazesi* were detected in positive Giemsa-stained blood films measuring 7.75 × 9.20 μm. Mature gametocytes appeared as a distinct parasite stage (Figure 1), occupying the entire cellular space and replacing the cell cytoplasm, occasionally forming elongated “horns.” Macrogametocytes appeared darker, with small nuclei, dark blue cytoplasm, light red nuclei, small vacuoles, and magenta volutin cytoplasmic granules. In contrast, microgametocytes showed lighter blue staining, with extremely pale cytoplasm and pale pink nuclei (Figure 2). Additionally, an average of 3 ± 0.4 *Leucocytozoon* sp. infected cells per field were recorded.

### 3.3 Gross lesions and histopathological findings

During necropsy, the examined pigeons typically exhibited no noticeable gross lesions. However, microscopic examination documented the presence of *Leucocytozoon* spp. in different organs, including the liver, spleen, and pancreas.

**Figure 3:**
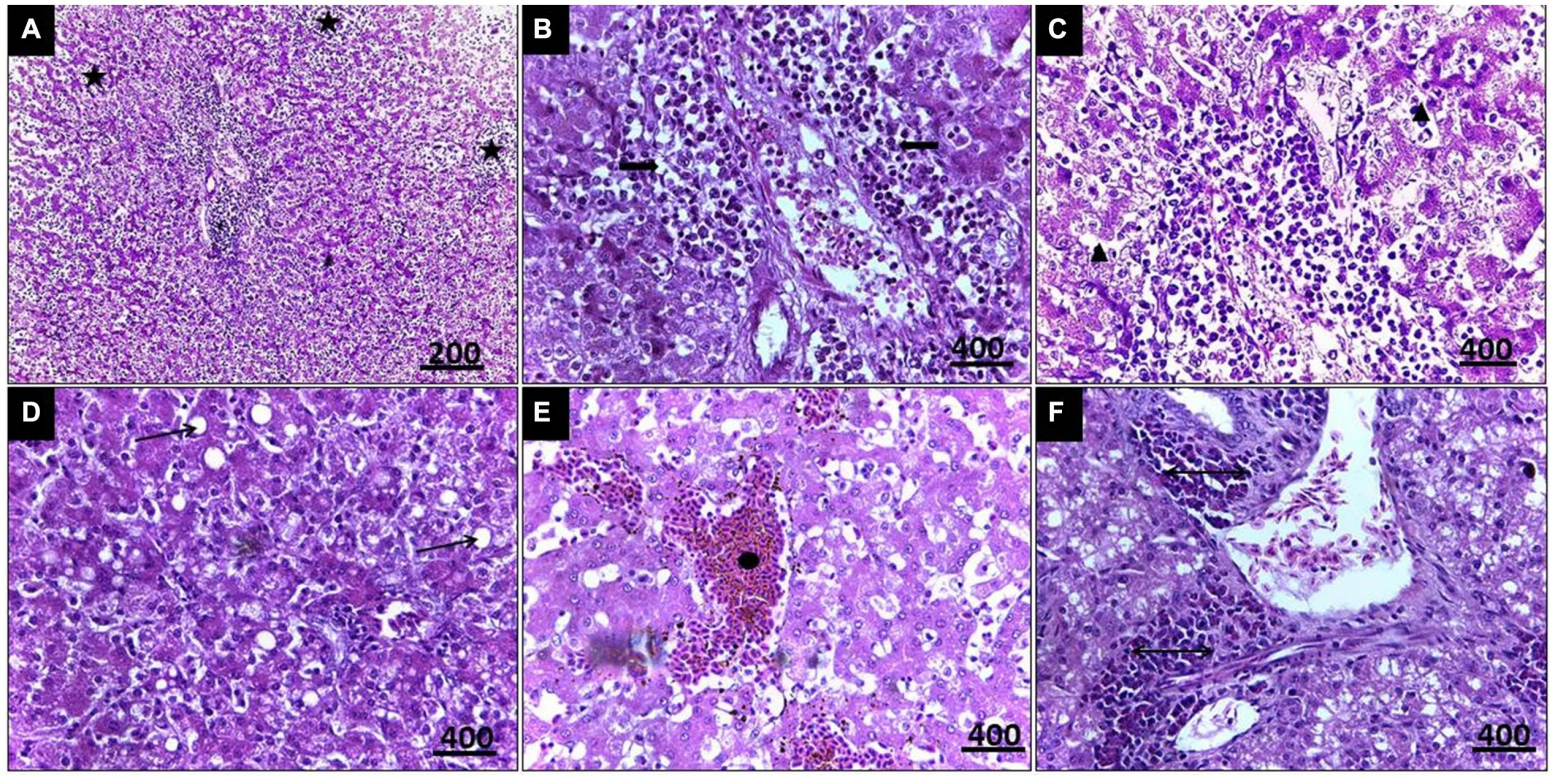
**(A)** Histopathological lesions of *Leucocytozoon* infection in a pigeon’s liver illustrate multiple necrosis foci (5-Point Star). **(B)** Numerous developing megaloschizont aggregations in the portal area (Left & Right Arrows). **(C)** Dilated blood sinusoids (Isosceles Triangle). **(D)** Fatty degeneration (Arrow). **(E)** Blood thrombosis (Oval). **(F)** Eosinophilic infiltration (Double arrow), H&E stain.

Histopathological examination of liver tissue (Figure 3) revealed focal areas of coagulative necrosis with distortion of hepatocytes. The periportal areas exhibited heavy infection with multiple variable-sized megaloschizonts containing numerous basophilic schizonts. These schizonts were observed solitarily or in groups, often surrounded by well-defined intact or depleted capsules. Additionally, there was notable infiltration of lymphocytes and macrophages around the schizonts’ periphery, contributing to hepatic cell necrosis near the portal areas. Other notable findings included portal vein congestion, blood sinusoid widening, fatty degeneration, and multiple thromboses in small and medium-sized hepatic vessels. Furthermore, congestion of the hepatic artery with thickening of its wall accompanied by amorphous eosinophilic infiltration was observed (Figure 3).

**Figure 4:**
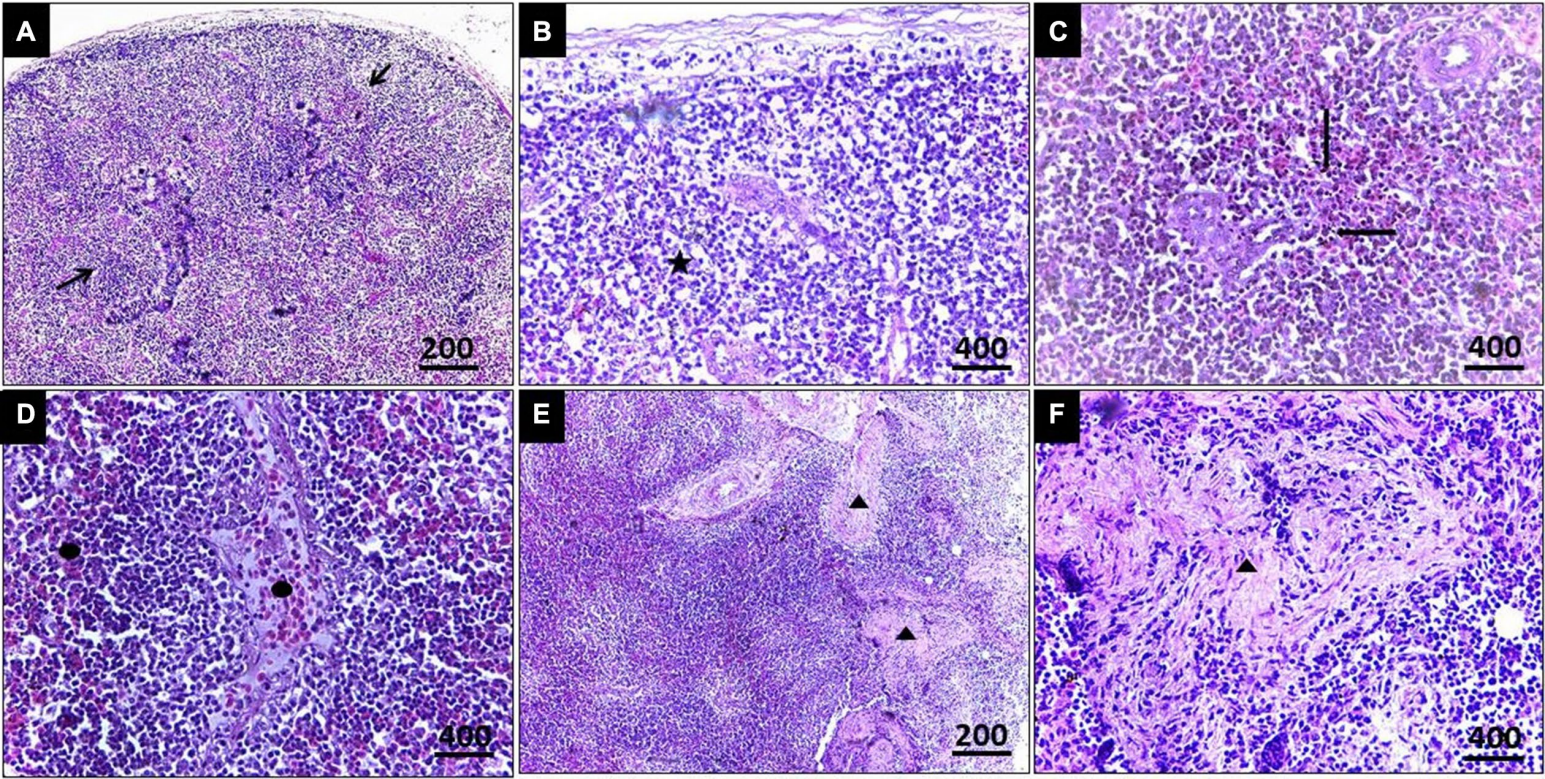
**(A)** Histopathological lesions of *Leucocytozoon* infection in the spleen of a pigeon illustrating necrotic areas with lymphocytic depletion (Arrows). **(B)** Few megaloschizonts distribution (5-Point Star). **(C)** Heamosedrosis (Left & down Arrows). **(D)** Lymphocytic cell infiltration and eosinophilic structures with vascular congestion (Oval). **(E,F)** Bands of fibrous tissue (Isosceles Triangle), H&E stain.

In the case of splenic infection (Figure 4), fewer megaloschizonts were observed in the interstitial tissue, accompanied by reticular hyperplasia. Intracellular hemosiderosis was noted as evidence for the destruction of erythrocytes. Moreover, lymphoid depletion and spleen atrophy with numerous eosinophilic structures (due to destructed megaloschizonts) were observed adjacent to the congested splenic artery. Bands of fibrous tissue were detected among splenic tissue.

**Figure 5:**
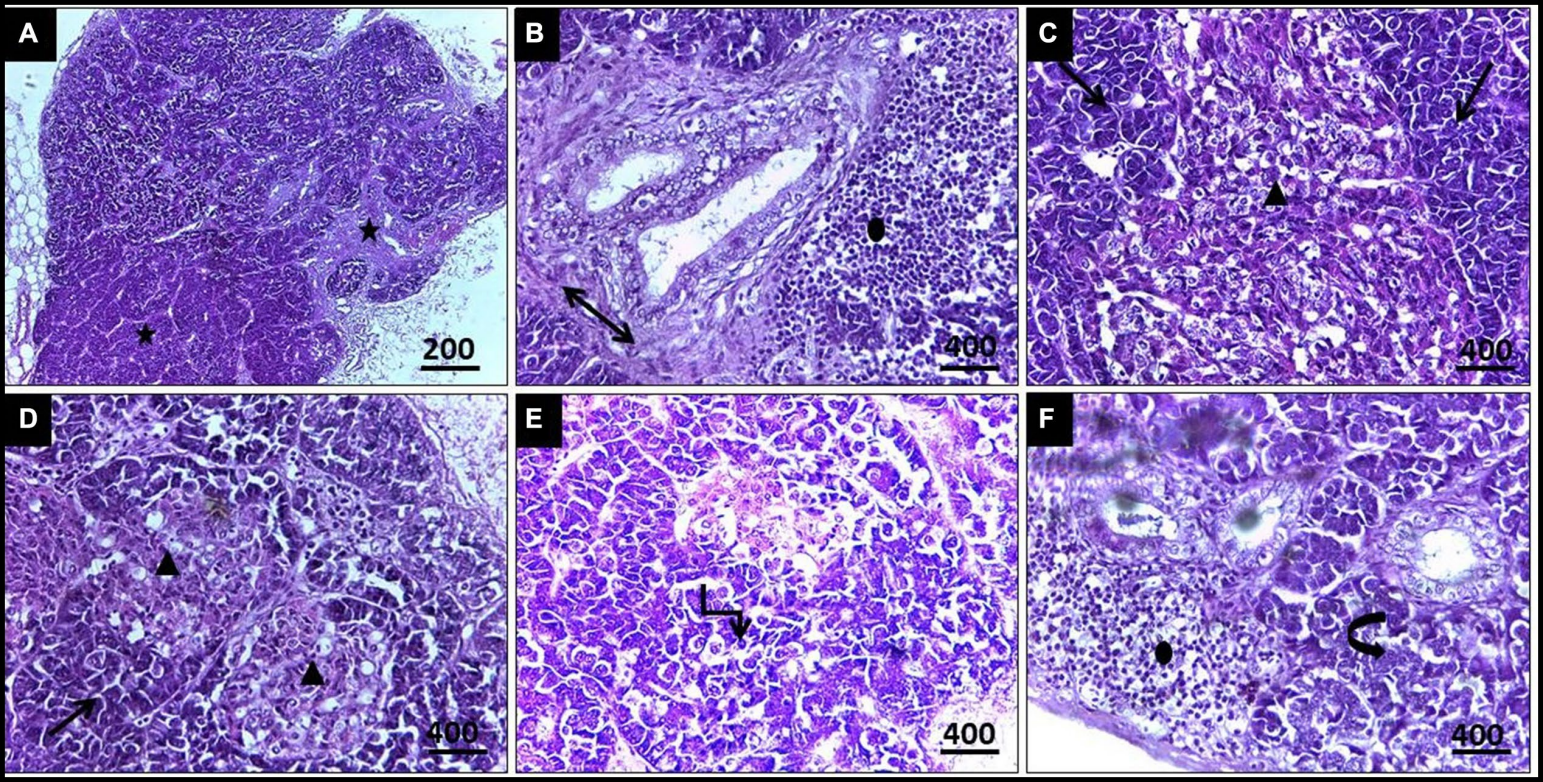
**(A,B)** Histopathological lesions of *Leucocytozoon* infection in a pigeon’s pancreas illustrate obvious necrosis areas with mild fibrous tissue proliferation (5-Point Star & Double Arrow). **(B)** Lymphocytic infiltration (Oval). **(C)** Numerous megaloschizonts (Isosceles Triangles). **(D)** Zymogen granules depletion (Arrows). **(E)** Apoptotic acinar cells (Elbow Arrow Connector). **(F)** Disorganized acinar cells (Curved Right Arrow), H&E stain.

Regarding histopathological lesions in the pancreas (Figure 5), acute pancreatic necrosis was observed, represented by zymogen granules depletion and shrinkage in the exocrine cells with degeneration in islets of Langerhans, resulting from megaloschizonts distribution. Bands of fibrous tissue were observed to separate the pancreatic ductular system, accompanied by multifocal mononuclear cell infiltrations consisting of lymphocytes and eosinophils. These features resulted in an apparent loss of acinar arrangement. Additionally, some acinar cells exhibited apoptotic vacuoles as a result of parasitism.

### 3.4 Molecular confirmation of *Leucocytozoon* by molecular methods

**Figure 6:**
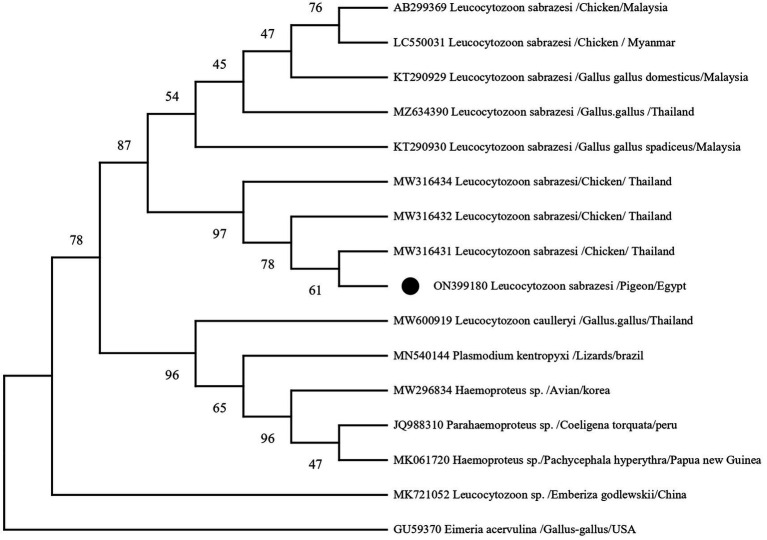
A phylogram of the maximum likelihood analysis of the *cytb* gene sequences of the *Leucocytozoon* spp. infecting poultry, depicting the robust association of the currently identified isolates of *Leucocytozoon sabrazesi* from pigeons marked by black circles (ON399180). The Maximum Likelihood Tree was implemented in MEGA software version 11 using *Eimeria acervulina* as outgroup. Bootstrap confident values were calculated with 500 repetitions.

In the present investigation, a single, homogenous electrophoretic band of 248 bp was yielded by polymerase chain reaction, resulting from the amplification of the mitochondrial DNA genome (*cytb* gene) within the nuclear ribosomal gene complex. Sequence analysis, depicted in Figure 6, revealed that all DNA sequences were identical to the *cytb* gene of *L. sabrazesi*. The obtained sequence was then deposited in GenBank with the accession number ON399180. BLASTN sequence analysis revealed 100% nucleotide sequence homology with the reference isolates. Additionally, nucleotide sequence homologies of 98.16, 95.85, and 93.55% were reported with sequences of *L. sabrazesi* isolates from chicken in Thailand (MW316432; MW316434) and Malaysia (AB299369), respectively (Figure 6).

## 4 Discussion

Leucocytozoonosis is a significant parasitic disease affecting avian hosts globally. This disease can lead to severe pathology and economic losses, including decreased meat yield and egg production. Previous literature has documented a limited number of reports on *Leucocytozoon* infection in various avian species in Africa (22, 23), New Zealand (24), and Turkey (25, 26). However, to our knowledge, no previous investigations have explored this parasite among pigeons in Egypt. Therefore, this study is the first to examine *Leucocytozoon* species’ prevalence and phenotypic characteristics among Egyptian pigeons.

The findings showed that 12.80% of examined birds were infected with *Leucocytozoon* species, highlighting the significant challenge of haemoparasitic infections in the studied area. This finding is consistent with the observation of Gocok et al. (26), who noted an infection rate of 13% for *Leucocytozoon* spp. among Turkish pigeons in Ankara province. However, a substantial disparity in the incidence of *Leucocytozoon* infection was observed compared to those recorded worldwide. Notably, very low prevalence rates of 2, 2.16, and 6.4% were reported among domestic pigeons from Bangladesh, India, and Nigeria, respectively (27–29). On the contrary, other surveys have reported higher infection rates, such as 30% in Pink pigeons (*Columba mayeri*) from the island of Mauritius in the Indian Ocean (30), 20% in pigeons from the Mymensingh district in Bangladesh (6), and 25% in various bird species from Europe, Africa, and North America (12). The significant fluctuations observed among the prevalence rates could be attributed to various factors, including differences in geographical locations, climatic conditions, bird breeds, management practices, sample sizes, detection methods employed, the presence of vectors, and study design (31–34).

The current investigation showed a significantly higher incidence of infection in adult birds than in young squabs, corroborating the findings of Garvin and Greiner (35). The same observation was documented in previous reports, revealing a significantly higher occurrence of avian haemoparasites in adults than grower birds in Bangladesh and Ethiopia (36, 37). However, our findings contradict previous reports from Kenya and Pakistan, which indicated that grower birds had a higher prevalence of avian haemoparasites than older birds (38, 39). Likewise, Van Oers et al. (40) and Castro et al. (41) observed a noticeably higher prevalence in young birds than in other age categories. On the other hand, other surveys concerned with blood parasites in various avian species have not found any significant association between the infection rate and host age (41–44). The positive correlation between the prevalence and age of screened birds could be accounted for higher mortality rates among young birds (2), declined immune response of adults (45), or increased exposure to the vectors (9).

The present results showed that the prevalence of *Leucocytozoon* spp. is influenced by sex, with a significantly higher infection rate in males (33.33%) than females (10.43%). This observation was supported by various previous reports (6, 46, 47). This observation is consistent with previous discussions by several authors who have suggested that higher testosterone levels and factors like stress during the courtship period play a significant role in immunosuppression in males, rendering them more susceptible to infection (48, 49). In contrast, Krone et al. (50) and Nath et al. (27) suggested that the highest peak of *Leucocytozoon* spp. prevalence rate is in female birds as compared to male pigeons.

According to the present findings, the incidence of *Leucocytozoon* parasite infection in pigeons in winter was the lowest (0%), and the difference between the winter and other seasons was statistically significant. Similarly, Nath and Bhuiyan (51) in Bangladesh demonstrated that the incidence of *Leucocytozoon* infections in pigeons during the summer was 60.6%, significantly lower in other seasons. Additionally, other studies on weaver birds of South Africa (52) and rock pigeons in India by Gupta et al. (53) were in the same line and documented that the summer season had the highest peak as compared to other seasons. However, the present finding disagrees with Senlik et al. (54), who demonstrated that the highest infection rate was recorded in the autumn season (44%), while the lowest rate was observed in the spring season in Iran. Moreover, Lawal et al. (55) revealed that the highest infection rates of haemoparasites, including *Leucocytozoon* spp., occurred during the rainy season (39.3%), followed by the cold dry (12.5%) season and the hot, dry season (7.7%). The potential explanation for the higher prevalence of *Leucocytozoon* in the dry season could be attributed to the warm climatic conditions that support the abundance of vector, *Simuliid* blackflies, which are widely distributed throughout the surrounding environment during this time of year (56–58).

In the current study, histopathological exploration revealed various lesions caused by *Leucocytozoon* spp. in the liver, spleen, and pancreas, including necrotic foci and loss of normal organization. Numerous megaloschizonts infiltrated the interstitial spaces, some displaying intact capsules with nuclei while others showed signs of degeneration. Surrounding the distribution of schizonts, a lymphocytic reaction with eosinophilic structures was observed, indicating a host defense response. Fatty degeneration was evident in the liver, indicating *Leucocytozoon* infection, pronounced hemosiderosis, lymphoid depletion in the spleen, and depletion of zymogen granules in the pancreas. Additionally, vascular congestion and thrombosis were diagnosed in some cases. Histopathology proved to be a definitive diagnostic tool for *Leucocytozoon* tissue reaction, with numerous megaloschizonts detected in highly vascularized organs such as the pancreas, lungs, pectoral muscles, liver, spleen, and heart. Our findings align with previously reported worldwide studies (59–61).

It should be noted that one of the most characteristic pathological lesions associated with *Leucocytozoon* infections includes fatty degeneration, which may sometimes be mistaken for fatty liver haemorrhagic syndrome. However, in cases of *Leucocytozoon* infection, distinct megaloschizonts are observed in various organs (62). Additionally, microscopic examination of spleens invaded by *Leucocytozoon* documented chronic inflammation characterized by aggregations of mononuclear cells and disorganized tissue with unclear boundaries between splenic pulps, resulting from merozoite invasion of erythrocytes (63). Furthermore, *Leucocytozoon* spp. colonization, blockage, and thrombosis lead to multiple focal areas of necrosis and ischemia, followed by cardio-respiratory failure and death (64).

Interestingly, our study marks the first molecular characterization of *Leucocytozoon* parasites in domestic pigeons from Egypt, shedding light on the presence of this parasite in avian hosts and expanding our understanding of blood parasites infecting Egyptian pigeons, addressing gaps in their phylogeny. Through sequencing analysis, we identified *Leucocytozoon sabrazesi* in pigeons. Furthermore, the isolate recovered in our study exhibited a 98.62% similarity to reference sequences (MW316431) previously identified in chickens. Similarly, research by Chawengkirttikul et al. (3) indicated a low diversity of *L. sabrazesi* populations in Thailand, with similarity values ranging from 89.5 to 100% with sequences from Malaysia and Myanmar. Additionally, genetic diversity studies of *Leucocytozoon* sp. based on *cytb* gene sequences have been conducted in various countries (7, 10, 23, 65–68), highlighting the *cytb* gene’s utility as an effective marker for phylogenetic taxonomy on a large scale and as a valuable tool for epidemiological analysis of leucocytozoonosis. Another survey in Egypt (11) reported the presence of *L. caulleryi* in broiler chicken flocks for the first time, showing a 99.14% similarity to strains recovered from Asian isolates in India, Japan, Malaysia, South Korea, Taiwan, and Thailand.

## 5 Conclusion

The current investigation marks the inaugural molecular study of haemosporidian parasites in pigeons in Egypt. Notably, our study stands as the pioneering genetic characterization of *L. sabrazesi* infection among pigeons, marking a national and global milestone. Additionally, our study has uncovered a significant statistical association between the infection prevalence of the parasite and various potential epidemiological factors, such as the age and sex of screened birds, with notable seasonal fluctuations observed throughout the year. Further studies are suggested to explore potential vectors at the national level, aiming to identify optimal preventive and therapeutic strategies against leucocytozoonosis, thereby mitigating or eradicating its detrimental impact on the bird industry. Additional large-scale surveys about the occurrence of the parasite within the avifauna of other regions in Egypt could provide valuable insights into blood parasite–host relationships and distribution patterns.

## Data availability statement

The original contributions presented in the study are included in the article/supplementary material, further inquiries can be directed to the corresponding author/s.

## Ethics statement

The animal studies were approved by the Ethics Committee, Faculty of Veterinary Medicine, South Valley University, Egypt according to the ethical regulations and guidelines for using animals in research (under permit code No. 84). Written and oral informed consent was obtained from the owners for the participation of their animals in this study. The studies were conducted in accordance with the local legislation and institutional requirements. Written informed consent was obtained from the owners for the participation of their animals in this study.

## Author contributions

IE: Conceptualization, Data curation, Formal analysis, Investigation, Methodology, Project administration, Software, Supervision, Validation, Visualization, Writing – original draft, Writing – review & editing. EM: Conceptualization, Data curation, Formal analysis, Investigation, Methodology, Project administration, Software, Visualization, Writing – original draft, Writing – review & editing. AM: Conceptualization, Data curation, Formal analysis, Investigation, Methodology, Software, Supervision, Validation, Visualization, Writing – original draft, Writing – review & editing. DS: Conceptualization, Data curation, Formal analysis, Investigation, Methodology, Project administration, Software, Validation, Visualization, Writing – original draft, Writing – review & editing. EA: Conceptualization, Data curation, Formal analysis, Investigation, Methodology, Project administration, Software, Validation, Visualization, Writing – original draft, Writing – review & editing. AA: Data curation, Formal analysis, Funding acquisition, Investigation, Resources, Software, Validation, Visualization, Writing – review & editing. HA: Data curation, Formal analysis, Funding acquisition, Investigation, Resources, Software, Validation, Writing – review & editing. EE: Conceptualization, Data curation, Formal analysis, Funding acquisition, Investigation, Methodology, Resources, Software, Validation, Visualization, Writing – original draft, Writing – review & editing.

## Footnotes

^1^www.totallab.com, Ver. 1.0.1

## References

[1] Attia MM Salem HM. Morphological and molecular characterization of *Pseudolynchia canariensis* (Diptera: Hippoboscidae) infesting domestic pigeons. Int J Trop Insect Sci. (2022) 42:733–40. doi: 10.1007/S42690-021-00597-2

[2] Valkiunas G. Avian malaria parasites and other Haemosporidia, vol. 1. Boca Raton, FL: CDC Press (2004).

[3] Chawengkirttikul R Junsiri W Watthanadirek A Poolsawat N Minsakorn S Srionrod N. Molecular detection and genetic diversity of Leucocytozoon sabrazesi in chickens in Thailand. Sci Rep. (2021) 11:16686. doi: 10.1038/S41598-021-96241-7, PMID: 34404893 PMC8370975

[4] Valkiunas G Iezhova TA. Exo-erythrocytic development of avian malaria and related haemosporidian parasites. Malar J. (2017) 16:101. doi: 10.1186/S12936-017-1746-7, PMID: 28253926 PMC5335495

[5] Telford SR. The hemoparasites of the reptilian: Color atlas and text. Boca Raton: CRC Press (2009).

[6] Dey A Begum N Anisuzzaman A Khan M Mondal M. Haemoprotozoan infection in ducks: prevalence and pathology. Bangl J Vet Med. (1970) 6:53–8. doi: 10.3329/BJVM.V6I1.1339

[7] Lee HR Koo BS Jeon EO Han MS Min KC Lee SB. Pathology and molecular characterization of recent *Leucocytozoon caulleryi* cases in layer flocks. J Biomed Res. (2016) 30:517–24. doi: 10.7555/JBR.30.2016K0017, PMID: 27760890 PMC5138585

[8] Ortego J Cordero PJ. PCR-based detection and genotyping of haematozoa (Protozoa) parasitizing eagle owls, *Bubo bub*. Parasitol Res. (2009) 104:467–70. doi: 10.1007/S00436-008-1207-X, PMID: 18818949

[9] Zhao W Pang Q Xu R Liu J Liu S Li J. Monitoring the prevalence of *Leucocytozoon sabrazesi* in southern China and testing tricyclic compounds against gametocytes. PLoS One. (2016) 11:e0161869. doi: 10.1371/JOURNAL.PONE.0161869, PMID: 27571513 PMC5003344

[10] Suprihati E Yuniarti WM. The phylogenetics of Leucocytozoon caulleryi infecting broiler chickens in endemic areas in Indonesia. Vet World. (2017) 10:1324–8. doi: 10.14202/VETWORLD.2017.1324-1328, PMID: 29263593 PMC5732337

[11] Elbestawy AR Ellakany HF Abd El-Hamid HS Gado AR Geneedy AM Noreldin AE. *Leucocytozoon caulleryi* in broiler chicken flocks: clinical, hematologic, histopathologic, and molecular detection. Avian Dis. (2021) 65:407–13. doi: 10.1637/0005-2086-65.3.407, PMID: 34427415

[12] Valkiunas G Iezhova TA Križanauskiene A Palinauskas V Sehgal RNM Bensch S. A comparative analysis of microscopy and PCR-based detection methods for blood parasites. J Parasitol. (2008) 94:1395–401. doi: 10.1645/GE-1570.1 18576856

[13] Soulsby EJL. Helminth, arthropods and Protozoa of domesticated animals. 7th Edn. Baillire, Tindall, 35–740. (1982).

[14] Levine ND. Veterinary protozoology. 1st Edn. Ames: Iowa State University Press, pp. 266–282. (1985).

[15] Madkour FA Abdelsabour-Khalaf M. Morphological and ultrastructural features of the laryngeal mound of Egyptian cattle egret (*Bubulcus ibis*, Linnaeus, 1758). BMC Zool. (2022) 7:44. doi: 10.1186/s40850-022-00147-4, PMID: 37170377 PMC10127301

[16] Chand N Faheem H Khan RU Qureshi MS Alhidary IA Abudabos AM. Anticoccidial effect of mananoligosacharide against experimentally induced coccidiosis in broiler. Environ Sci Pollut Res Int. (2016) 23:14414–21. doi: 10.1007/S11356-016-6600-X, PMID: 27068898

[17] Bancroft J D Stevens A. Theory and practice of histological techniques. 4th Edn. Edinburgh: Churchill Livingstone, pp. 273–292. (1996).

[18] Altschul SF Gish W Miller W Myers EW Lipman DJ. Basic local alignment search tool. J Mol Biol. (1990) 215:403–10. doi: 10.1016/S0022-2836(05)80360-2 2231712

[19] Thompson CE Worthington R Atkinson DR. Counselor content orientation, counselor race, and black Women's cultural mistrust and self-disclosures. J Couns Psychol. (1994) 41:155–61. doi: 10.1037/0022-0167.41.2.155

[20] Tamura K Stecher G Kumar S. MEGA11: molecular evolutionary genetics analysis version 11. Mol Biol Evol. (2021) 38:3022–7. doi: 10.1093/MOLBEV/MSAB120, PMID: 33892491 PMC8233496

[21] Serra-Freire NM. Planning and analysis for Parasitologic research. Niteroi: EdUFF (2002).

[22] Permin A Esmann JB Hoj CH Hove T Mukaratirwa S. Ecto-, endo- and haemoparasites in free-range chickens in the Goromonzi District in Zimbabwe. Prev Vet Med. (2002) 54:213–24. doi: 10.1016/S0167-5877(02)00024-7, PMID: 12114010

[23] Sehgal RNM Hull AC Anderson NL Valkiunas G Markovets MJ Kawamura S. Evidence for cryptic speciation of *Leucocytozoon* spp. (Haemosporida, Leucocytozoidae) in diurnal raptors. J Parasitol. (2006) 92:375–9. doi: 10.1645/GE-656R.1, PMID: 16729697

[24] Özmen Ö Haligür M Yukari BA. A study on the presence of leucocytozoonosis in wild birds of Burdur. Turk J Vet Anim Sci. (2005) 29:1273–8.

[25] Özmen Ö Halıgür M. Adanır RIdentification of different protozoa species from a common buzzard (*Buteo buteo*). Turk J Vet Anim Sci. (2009) 33:257–60. doi: 10.3906/vet-0803-29

[26] Gicik Y Arslan MO. Blood parasites of wild pigeons in Ankara district. Turk J Vet Anim Sci. (1999) 25:169–72.

[27] Nath TC Bhuiyan MJU Alam MS. A study on the presence of leucocytozoonosis in pigeon and chicken of hilly districts of Bangladesh. Issues Bio Sci Pharma Res. (2014) 2:13–8.

[28] Saikia M Bhattacharjee K Sarmah C Deka DK Tamuly S Kakati P. Prevalence and molecular detection of blood Protozoa in domestic pigeon. Int J Curr Microbiol Appl Sci. (2019) 8:1426–36. doi: 10.20546/ijcmas.2019.805.163

[29] Natala AJ Asemadahun ND Okubanjo OO Ulayi BM Owolabi YH Jato ID. A survey of parasites of domesticated pigeon (*Columba livia* domestica) in Zaria, Nigeria. Int J Soft Comput. (2009) 4:148–50.

[30] Swinnerton KJ Greenwood AG Chapman RE Jones CG. The incidence of the parasitic disease trichomoniasis and its treatment in reintroduced and wild pink pigeons *Columba mayeri*. Ibis. (2005) 147:772–82. doi: 10.1111/J.1474-919X.2005.00466.X

[31] La Chapelle M Ruta M Dunn JC. Bird species with wider geographical ranges have higher blood parasite diversity but not prevalence across the African-Eurasian flyway. Int J Parasitol. (2023) 53:787–96. doi: 10.1016/j.ijpara.2023.06.002, PMID: 37467874

[32] Valkiūnas G Iezhova TA. Insights into the biology of Leucocytozoon species (Haemosporida, Leucocytozoidae): why is there slow research Progress on agents of leucocytozoonosis? Microorganisms. (2023) 11:1251. doi: 10.3390/microorganisms11051251, PMID: 37317225 PMC10221462

[33] Sol D Jovani R Torres J. Geographical variation in blood parasites in feral pigeons: the role of vectors. Ecography. (2000) 23:307–14. doi: 10.1111/j.1600-0587.2000.tb00286.x

[34] Fecchio A Bell JA Bosholn M Vaughan JA Tkach VV Lutz HL. An inverse latitudinal gradient in infection probability and phylogenetic diversity for Leucocytozoon blood parasites in New World birds. J Anim Ecol. (2020) 89:423–35. doi: 10.1111/1365-2656.13117 31571223

[35] Garvin MC Greiner EC. Epizootiology of Haemoproteus danilewskyi (Haemosporina: Haemoproteidae) in blue jays (*Cyanocitta cristata*) in southcentral Florida. J Wildl Dis. (2003) 39:1–9. doi: 10.7589/0090-3558-39.1.1, PMID: 12685063

[36] Momin MA Begum N Dey AR Paran MS Zahangir AM. Prevalence of blood protozoa in poultry in Tangail, Bangladesh. IOSR J Agric Vet Sci. (2014) 7:55–60. doi: 10.9790/2380-07735560

[37] Etisa E Chanie M Tolossa YH. Prevalence of Haemoparasites infections in scavenging indigenous chickens in and around Bishoftu. World Appl Sci J. (2017) 35:302–9. doi: 10.5829/idosi.wasj.2017.302.309

[38] Sabuni ZA Mbuthia PG Maingi N Nyaga PN Njagi LW Bebora LC. Prevalence of haemoparasites infection in indigenous chicken in Eastern Province of Kenya. Livest Res Rural Dev. (2011) 23:11.

[39] ul H NMA Khan MK Iqbal Z Rizwan HM Khan MN Naqvi SZ. Prevalence and associated risk factors of haemoparasites, and their effects on hematological profile in domesticated chickens in district Layyah, Punjab, Pakistan. Prev Vet Med. (2017) 143:49–53. doi: 10.1016/J.PREVETMED.2017.05.001, PMID: 28622791

[40] Van Oers K Richardson DS Sæther SA Komdeur J. Reduced blood parasite prevalence with age in the Seychelles warbler: selective mortality or suppression of infection? J Ornithol. (2010) 151:69–77. doi: 10.1007/S10336-009-0427-X

[41] Castro I Howe L Tompkins DM Barraclough RK Slaney D. Presence and seasonal prevalence of plasmodium spp. in a rare endemic New Zealand passerine (tieke or saddleback, *Philesturnus carunculatus*). J Wildl Dis. (2011) 47:860–7. doi: 10.7589/0090-3558-47.4.860, PMID: 22102656

[42] Kučera V. New results in state estimation and regulation. Automatica. (1981) 17:745–8. doi: 10.1016/0005-1098(81)90021-2

[43] Hauptmanova K Maly M Literak I. Changes of haematological parameters in common pheasant throughout the year. Vet Med (Praha). (2006) 51:29–34. doi: 10.17221/5514-VETMED

[44] Scaglione FE Pregel P Cannizzo FT Pérez-Rodríguez AD Ferroglio E Bollo E. Prevalence of new and known species of haemoparasites in feral pigeons in Northwest Italy. Malar J. (2015) 14:99. doi: 10.1186/S12936-015-0617-3, PMID: 25888761 PMC4350268

[45] Cichoń M Sendecka J Gustafsson L. Age-related decline in humoral immune function in collared flycatchers. J Evol Biol. (2003) 16:1205–10. doi: 10.1046/J.1420-9101.2003.00611.X, PMID: 14640412

[46] Opara MN Ogbuewu IP Njoku L Ihesie EK Etuk IF. Study of the haematological and biochemical values and gastrointestinal and haemoparasites in racing pigeons (*Columba livia*) in Owerri, Imo state. Niger. Rev. Cient. UDO Agríc. (2012) 12:955–9.

[47] Hussein NM Abdelrahim EA. *Haemoproteus columbae* and its histopathological effects on pigeons in Quena governorate, Egypt. IOSR J Pharm Biol Sci. (2016) 11:79–90. doi: 10.9790/3008-11117990

[48] Folstad I Karter AK. Parasites, bright males and the immunocompetence handicap. Am. Nat. (1992) 139:603–22. doi: 10.1086/285346

[49] Zuk M McKean KA. Sex differences in parasite infections: patterns and processes. Int J Parasitol. (1996) 26:1009–24. doi: 10.1016/S0020-7519(96)80001-4 8982783

[50] Krone O Priemer J Streich J Sommer P Langgemach T Lessow O. Haemosporida of birds of prey and owls from Germany. Acta Protozool. (2001) 40:281–90.

[51] Nath TC Bhuiyan JU. Haemoprotozoa infection of domestic birds in hilly areas of Bangladesh. Indep J Manag Prod. (2017) 8:82–90. doi: 10.14807/ijmp.v8i1.520

[52] Okanga S Cumming GS. Avian malaria prevalence and mosquito abundance in the Western cape, South Africa. Malar J. (2013) 12:370. doi: 10.1186/1475-2875-12-370, PMID: 24160170 PMC4016263

[53] Gupta DK Jahan N Gupta N. New records of Haemoproteus and plasmodium (Sporozoa: Haemosporida) of rock pigeon (*Columba livia*) in India. J Parasit Dis. (2011) 35:155–68. doi: 10.1007/S12639-011-0044-5, PMID: 23024498 PMC3235393

[54] Şenlik B Gulegen E Akyol V. Prevalance and intensity of *Haemoproteus columbae* in domestic pigeons. Indian Vet J. (2005) 28:998–9. 10.1556/AVet.53.2005.4.5 16363146

[55] Lawal JR Ibrahim UI Biu AA Musa HI. Prevalence and risk factors associated with Haemoparasitosis in village chickens (*Gallus gallus* Domesticus) in Gombe state, Nigeria. Vet Sci Res. (2019) 4:000190:1–14. doi: 10.23880/oajvsr-16000190

[56] Otabil KB Gyasi SF Awuah E Obeng-Ofori D Tenkorang SB Kessie JA. Biting rates and relative abundance of Simulium flies under different climatic conditions in an onchocerciasis endemic community in Ghana. Parasit Vectors. (2020) 13:1–10. doi: 10.1186/S13071-020-04102-5/FIGURES/7 32375902 PMC7204027

[57] Walsh KJE Ryan BF. Tropical cyclone intensity increase near Australia as a result of climate change. J Clim. (2000) 13:3029–36. doi: 10.1175/1520-0442(2000)013<3029:TCIINA>2.0.CO;2

[58] Zamora-Vilchis I Williams SE Johnson CN. Environmental temperature affects prevalence of blood parasites of birds on an elevation gradient: implications for disease in a warming climate. PLoS One. (2012) 7:e39208. doi: 10.1371/journal.pone.0039208, PMID: 22723966 PMC3378574

[59] Omori S Sato Y Toda H Sasaki K Isobe T Nakanishi T. Use of flow cytometry to separate *Leucocytozoon caulleryi* gametocytes from avian blood. Parasitology. (2010) 137:1899–903. doi: 10.1017/S0031182010000880 20619066

[60] Suprihati E Kusnoto K Triakoso N Yuniarti WM. Histopathological studies on *Leucocytozoon caulleryi* infection on broiler in endemic area of Indonesia. Sys Rev Pharm. (2020) 11:1219–23. doi: 10.31838/SRP.2020.11.175

[61] Win SY Chel HM Hmoon MM Htun LL Bawm S Win MM. Detection and molecular identification of Leucocytozoon and plasmodium species from village chickens in different areas of Myanmar. Acta Trop. (2020) 212:105719. doi: 10.1016/J.ACTATROPICA.2020.105719, PMID: 32976841

[62] Crespo R Shivaprasad H. Developmental, metabolic, and other noninfectious disorders[a] In: Swayne D Glisson J McDougald L Nolan L Suarez D Nair V, editors. Diseases of poultry[M]. 13th ed. Aimes: Wiley-Blackwell (2013). 1233–70.

[63] Ohnishi Y Nishimura K. Role of reticulocytes on gametocytogenesis in chickens infected with *Leucocytozoon caulleryi*. J Vet Med Sci. (2001) 63:797–800. doi: 10.1292/JVMS.63.797, PMID: 11503908

[64] Donovan TA Schrenzel M Tucker TA Pessier AP Stalis IH. Hepatic hemorrhage, hemocoelom, and sudden death due to *Haemoproteus* infection in passerine birds: eleven cases. J Vet Diagn Invest. (2008) 20:304–13. doi: 10.1177/104063870802000307, PMID: 18460616

[65] Sato Y Tamada A Mochizuki Y Nakamura S Okano E Yoshida C. Molecular detection of *Leucocytozoon lovati* from probable vectors, black flies (Simuliudae) collected in the alpine regions of Japan. Parasitol Res. (2009) 104:251–5. doi: 10.1007/S00436-008-1183-1, PMID: 18791737

[66] Murdock CC Adler PH Frank J Perkins SL. Molecular analyses on host-seeking black flies (Diptera: Simuliidae) reveal a diverse assemblage of Leucocytozoon (Apicomplexa: Haemospororida) parasites in an alpine ecosystem. Parasit Vectors. (2015) 8:952. doi: 10.1186/S13071-015-0952-9 PMC4486084 26108211

[67] Argilla LS Howe L Gartrell BD Alley MR. High prevalence of Leucocytozoon spp. in the endangered yellow-eyed penguin (*Megadyptes antipodes*) in the sub-Antarctic regions of New Zealand. Parasitology. (2013) 140:672–82. doi: 10.1017/S0031182012002089, PMID: 23361092

[68] Galen SC Nunes R Sweet PR Perkins SL. Integrating coalescent species delimitation with analysis of host specificity reveals extensive cryptic diversity despite minimal mitochondrial divergence in the malaria parasite genus Leucocytozoon. BMC Evol Biol. (2018) 18:1242. doi: 10.1186/S12862-018-1242-X PMC6117968 30165810

